# Associations between Two Genetic Variants in *NKX2-5* and Risk of Congenital Heart Disease in Chinese Population: A Meta-Analysis

**DOI:** 10.1371/journal.pone.0070979

**Published:** 2013-08-02

**Authors:** Zhenling Wang, Li Zou, Rong Zhong, Beibei Zhu, Wei Chen, Na Shen, Juntao Ke, Jiao Lou, Ranran Song, Xiao-Ping Miao

**Affiliations:** 1 Department of Epidemiology and Biostatistics and State Key Laboratory of Environment Health (Incubation), Ministry of Education Key Laboratory of Environment and Health, Ministry of Environmental Protection Key Laboratory of Environment and Health, School of Public Health, Tongji Medical College, Huazhong University of Science and Technology, Wuhan, China; 2 Department of Maternal and Child Health, School of Public Health, Tongji Medical College, Huazhong University of Science and Technology, Wuhan, China; New York Medical College, United States of America

## Abstract

**Background:**

NKX2-5 is a transcriptional factor, which plays an important role in heart formation and development. Two genetic variants in the coding region of *NKX2-5,* 63A>G (rs2277923) and 606G>C (rs3729753), have been investigated in the risk of congenital heart disease (CHD), although with inconsistent results. Thus, a meta-analysis was performed to clarify the associations between the two variants and CHD risk in the Chinese population.

**Methods and Results:**

Relevant studies were identified by searching PubMed, ISI Web of Science and CNKI databases and by reviewing the reference lists of retrieved articles. Then, the data from eligible studies were combined in an allelic model. A total of 7 and 4 studies were ultimately included for 63A>G and 606G>C, respectively. The results of overall meta-analyses showed that significant association was detected for 63A>G (OR = 1.26, 95% CI = 1.02–1.56, *P*_heterogeneity_ = 0.009, *I* ^2^ = 65.1%), but not for 606G>C (OR = 1.22, 95% CI = 0.75–1.96, *P*_heterogeneity_ = 0.412, *I* ^2^ = 0.0%). Regarding 63A>G variant, positive results were also obtained in the subgroups of atrial septal defect and large-sample-size study. Besides, the sensitivity analysis indicated that significant association was still detected after deletion of the individual studies with positive result and striking heterogeneity.

**Conclusion:**

Our results revealed that the 63A>G variant in *NKX2-5*, but not the 606G>C, may contribute to CHD risk for Chinese.

## Introduction

Congenital heart disease (CHD) is defined as a gross structural anomaly of the heart or intrathoracic great vessels that is actually or potentially of functional significance [1]. As the most common developmental abnormality, CHD has a incidence of approximately 9.1 in 1000 new born babies worldwide and the situation is more severe in premature, death and abortion forms [2]. According to the report provided by China’s Ministry of Health in 2012, the incidence of CHD during perinatal period was estimated to be 40.95/10,000 [3]. CHD contains 15 clinical types at least and its morbidity and mortality are significantly greater than that of the general individuals, even after effective surgical correction [4], [5]. Despite its high prevalence and poor prognosis, the causes of CHD are largely unknown. Accumulating evidence has indicated that genetic factor plays a crucial role in CHD, with high degree of heritability [6]. In 2012, a systematic review conducted by Shieh JT et al. further demonstrated that CHD risk significantly increased in consanguineous unions of the studied populations, principally at first-cousin level [7].

Cardiac transcription factors, such as NKX2-5, GATA4, TBX5 and TBX20, strictly regulate the development of heart through a regulatory network [8]–[10]. Recently, a growing number of variants in these genes have been found to implicate in CHD cases [11]–[13], suggesting their potentially important roles in the occurrence of CHD. Among these cardiac transcription factors, the *NKX2-5* gene, located at chromosome 5q34, was firstly identified to be involved in heart development of many organisms, such as zebrafish, frog, chicken, mouse and human being. A series of clinical manifestations for CHD including arrhythmia, cardiac contractility defects, cardiac structural defects and premature death were observed in *NKX2-5* knocked out mice [14], [15]. Besides, family studies revealed that widely variable expressivity of *NKX2-5* would also lead to CHD [16], [17]. Moreover, *NKX2-5* not only individually acts in transcriptional regulation, but also interacts with other transcription factors in the early stage of cardiac development [18]–[24]. A synonymous variant 63A>G (rs2277923, Glu21Glu) located in the exon 1 was reported to be associated with risk of CHD. In 1999, Benson et al. firstly detected the variant in 52 normal controls [25]. Moreover, the variant was also identified by Reamon-Buettner et al. in the diseased heart tissues of 68 patients with CHD by direct sequencing, but the difference in allele frequency was insignificant between CHD cases and healthy controls [26]. However, Shi et al. observed significant association in 110 case-control pairs of Chinese [27], which was followed by a number of studies trying to replicate the attractive result in the Chinese population, but with inconsistent findings [28]–[33]. Another synonymous variant 606G>C (rs3729753, Leu202Leu) located in the exon 2 of *NKX2-5*, was also suspected to implicate in CHD risk in the Chinese population. Based on the controversial conclusions and limited statistical power of individual studies, as well as almost all relevant data obtained from the Chinese population, we performed a meta-analysis of published studies to provide more precise estimations for the associations between 63A>G and 606G>C variants and the risk of CHD in Chinese population.

## Methods

### Identification and Eligibility of Studies

Relevant articles published up to February 2013 were identified by searching PubMed and ISI Web of Science databases using the key words “*NKX*/*CSX*” and “*congenital heart disease/defects*” without language restriction. To expand the coverage of our searches, we further performed search in China National Knowledge Infrastructure (CNKI) database applying the above key words. References of retrieved articles were also scanned. We conducted the meta-analysis and reported its results according to the Preferred Reporting Items for Systematic Reviews and Meta-Analysis (PRISMA) statement (Checklist S1).

We used following criteria to select the eligible studies: (1) case-control studies in the Chinese population; (2) evaluation of the *NKX2-5* 63A>G and/or 606G>C variant(s) and CHD risk; (3) non-syndromic CHD confirmed histologically or pathologically; (4) presentation of data necessary for calculating odds ratios (ORs) with 95% confidence intervals (CIs). All clinical types, such as atrial septal defect (ASD), ventricular septal defect (VSD), patent ductus arteriosus and patent formen ovale were included in this meta-analysis. Review, case report, simply commentary, animal study, unpublished report and the study with CHD being considered as a component of well-known genetic syndromes or a multiple congenital anomaly syndrome were excluded.

### Data Extraction

Data were extracted independently by two reviewers. The following information was extracted from the eligible studies: first author, year of publication, design type of study, types of CHD, source of DNA, whether Hardy-Weinberg equilibrium (HWE) accorded in control population and counts of alleles or genotypes in case and control groups.

### Statistical Analysis

Goodness-of-fit *x* ^2^ test was applied to assess HWE in controls if there were no definitive statements about whether the genotype frequencies in control groups were compatible with HWE in the original. The associations between *NKX2-5* 63A>G, 606G>C variants and risk of CHD were evaluated by allelic ORs and their 95% CIs because of only allele frequencies reported in some studies. The Cochran’s *Q* statistic test was employed to estimate the between-study heterogeneity [34]. When the *P* value of *Q* statistic test was <0.1, the random-effects model was used due to significant heterogeneity [35]; otherwise, the fixed-effects model was applied [36]. Overall meta-analysis was initially conducted and then stratified analysis, if feasible, was carried out according to the types of CHD and sample size. Additionally, sensitivity analysis was conducted, in which the pooled ORs were calculated after omission of each study in turn. Publication bias was assessed by funnel plot and Egger’s test [37]. The above analyses were conducted in Stata10.0 and all *P* values less than 0.05 were considered statistically significant for all tests except for *Q* test. Besides, the power analysis was carried out by Power V3.0 software (http://dceg.cancer.gov/tools/design/POWER).

## Results

### Characteristic of Included Studies

**Figure 1:**
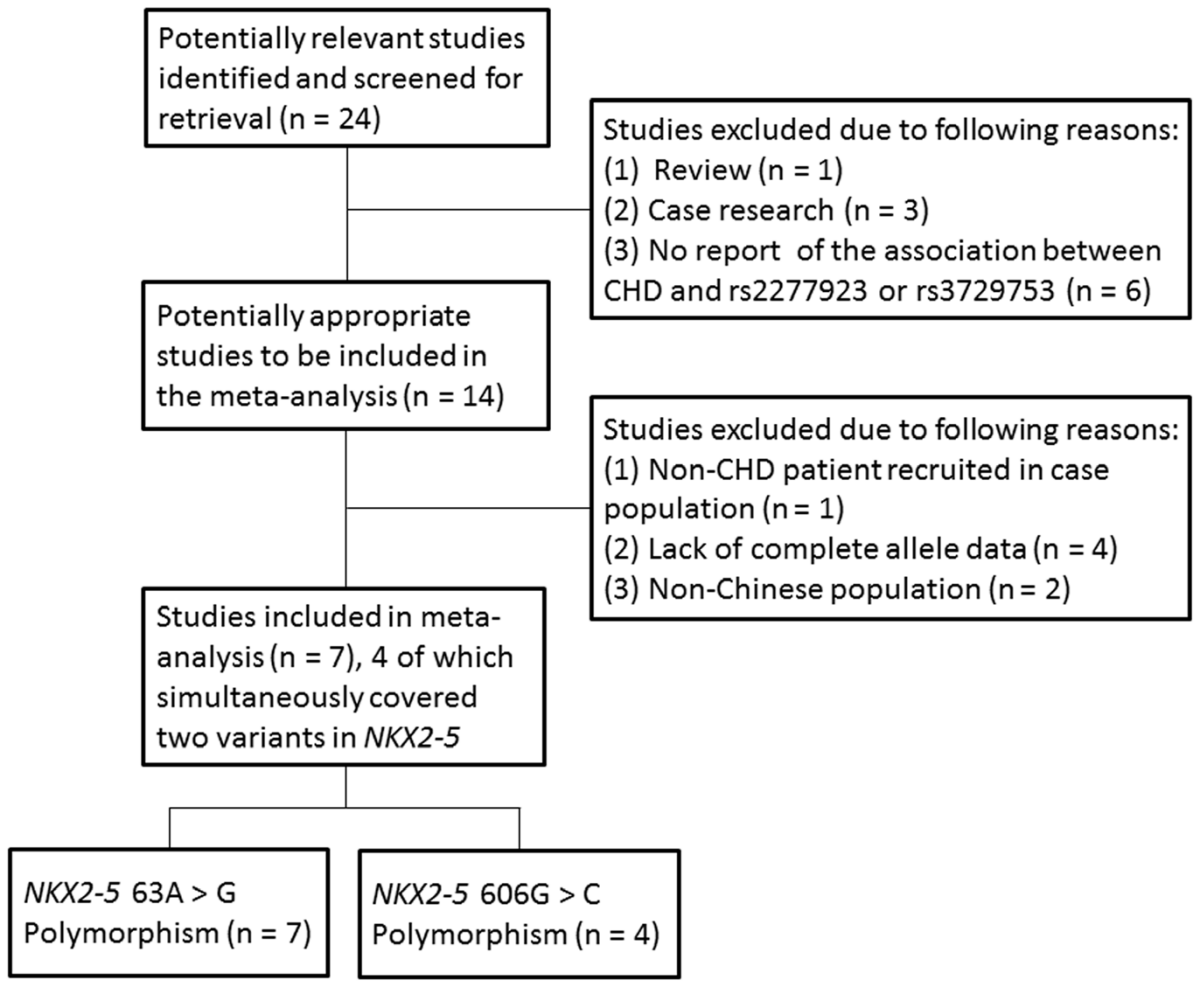
Flow diagram of the study selection procedure.

**Figure 1** shows the literature search and study selection procedures. Twenty-four articles were initially identified through the above search strategy. After review of the titles and abstracts, 10 articles were excluded. Seven publications were further excluded after review of the full texts because of the following reasons: the cases in the study by Ouyang et al. suffered from coronary artery disease (CAD) and rheumatic heart disease (RHD) [38]; two studies carried out in non-Chinese populations, plus one of which extracted DNA from formalin fixed tissue samples [26], [39]; no complete allele data obtained in other four studies [18], [40], [41], [42]. Finally, 7 studies containing 1243 cases and 1139 controls were relevant to 63A>G and 4 studies containing 748 cases and 630 controls were relevant to 606G>C. Of these studies, 3 studies just explored a single type of CHD [28], [29], [33], whereas multiple types of CHD were involved in the other studies [27], [30], [31], [32]. A same set of control was applied across two studies by Liu et al [28], [29]. Besides, whether the control group in the study by Xiong et al. accordant with HWE was neither mentioned in the original, nor calculated by goodness-of-fit *x* ^2^ test due to lack of genotype frequencies [32]. The characteristics of the included studies are shown in **Table 1**.

**Table 1:** Characteristics of included studies.

First author	Publication year	Country	Study type	Variants	Types of CHD	Case/control	DNA source	HWE
Shi	2005	China	Case-control	63A>G	Multiple	110/110	Blood	Y
Liu^b^	2009	China	Case-control	63A>G	ASD	180/200	Blood	Y
Liu^b^	2009	China	Case-control	63A>G 606G>C	VSD	160/200	Blood	Y
Zhang	2009	China	Case-control	63A>G 606G>C	Multiple	230/200	Blood	Y
Peng	2010	China	Case-control	63A>G 606G>C	Multiple	**63A>G:** 126/114**606G>C:** 134/109	Blood	Y
Xiong	2012	China	Case-control	63A>G606G>C	Multiple	224/121	Blood	–a
Pang	2012	China	Case-control	63A>G	VSD	213/194	Blood	Y

Abbreviations: HWE, Hardy-Weinberg equilibrium; CHD, congenital heart disease; ASD, atrial septal defect; VSD, ventricular septal defect.

a Not mentioned nor could be figured out.

b A same set of control applied across two case-control analyses.

### Overall Meta-analysis

**Figure 2:**
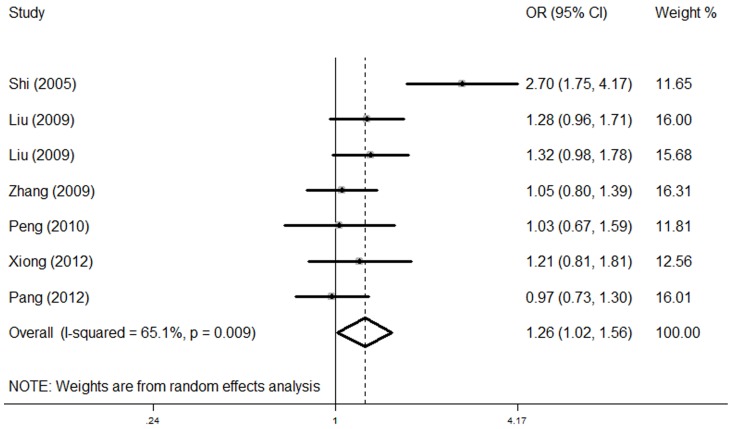
The forest plots of ln (OR) with 95% CIs for the *NKX2-5* 63A>G for CHD. Random-effects pooled OR = 1.26, 95% CI = 1.02–1.56, *P* = 0.034; *P*_heterogeneity_ = 0.009.

**Figure 3:**
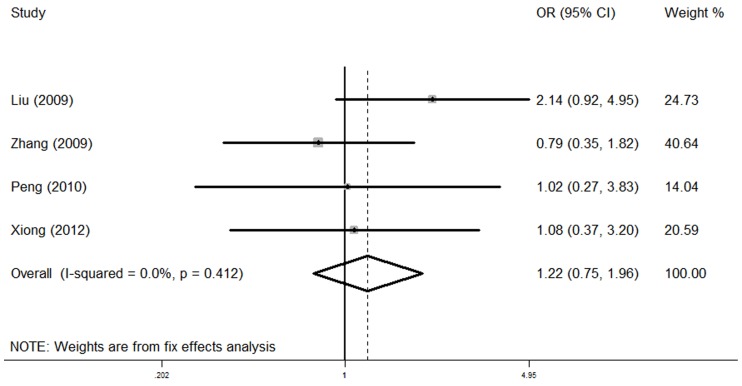
The forest plots of ln (OR) with 95% CIs for the *NKX2-5* 606G>C for CHD. Fixed-effects pooled OR = 1.22, 95% CI = 0.75–1.96, *P* = 0.422; *P*_heterogeneity_ = 0.412.

**Figure 2** shows the combined result for 63A>G in associated with CHD. A random-effects model was used because of significant heterogeneity (*P*_heterogeneity_ = 0.009, *I* ^2^ = 65.1%). Individuals with the *NKX2-5* 63G allele showed a significantly increased CHD risk compared with those with the A allele (OR = 1.26, 95% CI = 1.02–1.56, *P* = 0.034). For 606G>C, no substantial heterogeneity was detected (*P*_heterogeneity_ = 0.412). In the fixed-effects model, no significant association between this variant and CHD was found (OR = 1.22, 95% CI = 0.75–1.96, *P* = 0.422, *P*_heterogeneity_ = 0.412, *I* ^2^ = 0.0%, **Figure 3**). Besides, for *NKX2-5* A63G with minor allele frequency of 0.433, we calculated that the power for our sample size to detect an OR of 1.26 was 0.801; for *NKX2-5* G606C with minor allele frequency of 0.022, the power for our sample size to detect an OR of 1.22 was 0.079.

### Stratified Analysis

The data were firstly stratified by the types of CHD into two subgroups, the VSD and ASD subgroups. Significant association was observed between 63A>G and ASD (OR = 1.31, 95% CI = 1.00–1.71, *P* = 0.048, *P*_heterogeneity_ = 0.719, *I* ^2^ = 0.0%). However, no statistical evidence for the association between 63A>G and VSD was detected (OR = 1.67, 95% CI = 0.87–3.21, *P* = 0.124, *P*_heterogeneity_ = 0.000, *I* ^2^ = 89.5%, **Table 2**).

**Table 2:** Associations between *NKX2-5* 63A>G and CHD stratified by types of CHD and sample size.

Variables	No.^a^	Case/control	OR (95% CI)	I-square (%)	*P* for heterogeneity^b^
Type					
VSD	3	433/504	1.67 (0.87–3.21)	89.5%	*P* = 0.000
ASD	2	202/310	1.31 (1.00–1.71)	0.0%	*P* = 0.719
Sample size^c^					
Small	2	236/224	1.67 (0.65–4.27)	89.4%	*P* = 0.002
Large	5	1007/665	1.15 (1.01–1.32)	0.0%	*P* = 0.538

Abbreviations: OR, Odds ratio; CI, confidence interval.

a The number of articles.

b When *P* value of the heterogeneity test was >0.1, the fixed-effects model was used; otherwise, the random-effects model was applied.

c The study was regarded as large-sample-size study, if the number of case was greater than 150; otherwise, the study was defined as small-sample-size study.

The data were then stratified by sample size. The study was regarded as large-sample-size if the number of case is greater than 150; otherwise, the study was defined as small-sample-size [43], [44]. In the large-sample-size subgroup, significant association between 63A>G and CHD was detected (OR = 1.15, 95% CI = 1.01–1.32, *P* = 0.040, *P*_heterogeneity_ = 0.538, *I* ^2^ = 0.0%). While no significant risk of CHD associated with 63A>G was observed in the small-sample-size subgroup (OR = 1.67, 95% CI = 0.65–4.27, *P* = 0.285, *P*_heterogeneity_ = 0.002, *I* ^2^ = 89.4%, **Table 2**).

The association regarding 606G>C and CHD could not be assessed due to insufficient data.

### Sensitivity Analysis

Given the significant between-study heterogeneity, sensitivity analysis was carried out to assess the effect of each study on the overall estimate. The association between 63A>G and CHD turned to be marginal statistical significance when several studies were omitted. Besides, the heterogeneity was drastically reduced after deletion of the study by Shi et al. (*P*_heterogeneity_ = 0.648, *I* ^2^ = 0.0%)(**Table 3**). In terms of 606G>C, the sensitivity analysis demonstrated relatively robust results with no reverse outcome (**Table 3**).

**Table 3:** Sensitivity analysis of pooled studies for CHD on *NKX2-5* 63A>G and 606G>C.

Variant	Study omitted	OR (95% CI)	I-square (%)	*P* for heterogeneity^a^
63A>G	Shi	1.14 (1.00–1.30)	0.0	0.648
	Liu	1.26 (0.97–1.64)	70.7	0.004
	Liu	1.25 (0.97–1.62)	70.3	0.005
	Zhang	1.31 (1.02–1.68)	68.3	0.007
	Peng	1.30 (1.02–1.65)	69.8	0.005
	Xiong	1.27 (1.00–1.63)	70.9	0.004
	Pang	1.32 (1.05–1.68)	64.9	0.014
606G>C	Liu	0.91 (0.51–1.64)	0.0	0.890
	Zhang	1.51 (0.83–2.73)	0.0	0.507
	Peng	1.25 (0.75–2.08)	28.4	0.247
	Xiong	1.25 (0.74–2.13)	29.1	0.244

a When *P* value of the heterogeneity test was >0.1, the fixed-effects model was used. Otherwise, the random-effects model was used.

### Publication Bias

As reflected by funnel plots (**Figure S1 and Figure S2**) and Egger’s test, no publication bias was detected for 63A>G (*P* = 0.249) and 606G>C in *NKX2-5* (*P* = 0.797).

## Discussion

The current meta-analysis suggested that the 63A>G variant in *NKX2-5* was significantly associated with the risk of CHD in the Chinese population, whereas the 606 G>C did not appear to have an effect on CHD susceptibility. Besides, positive results with regard to 63A>G were found in the subgroups of ASD and large-sample-size study with no heterogeneity.

*NKX2-5* acts as a prominent candidate CHD-associated gene given its crucial role in heart morphogenesis and function. Embryonic lethality, growth retardation and abnormal heart morphogenesis were observed in mice with targeted *NKX2-5* disruption due to impaired cardiac looping [45]. *NKX2-5*, as the fifth gene identified in the *NK-2* homeobox gene family, consists of two exons which encode a 324-amino-acid protein [46], [47]. The protein encoded by *NKX2-5* is a transcription factor comprising homeodomain (HD), TN and NK2-specific (NK2-SD) domains. It is estimated that there are more than 33 variants detected in *NKX2-5* as yet, in which, the 63A>G and 606G>C polymorphsims studied most extensively were included in our meta-analysis [31], [48]. The 63A>G variant was found to be significantly associated with CHD risk in this meta-analysis. Synonymous variants are gradually acknowledged to alter protein expression, conformation and function through mechanisms of affecting splicing accuracy, translation fidelity, mRNA structure and protein folding [49]. It has reported that the 63A>G variant weakened the transactivation activity of *NKX2.5* by approximately 20% [38]. Furthermore, the RESCUE ESE, a program for validation of exonic splicing enhancers, demonstrated that 63A>G could repeal an exonic splice site starting 4 base pairs upstream of the variant [50], exerting an influence on translational kinetics. Although there are some evidence for the role of 63A>G variant in CHD, the underlying mechanism is required further investigation. Alternatively, we cannot rule out the possibility of its involvement to linkage disequilibrium (LD) with other disease causing variants. For another variant 606G>C, no promising association was found in this meta-analysis, which was consistent with multiple studies [29], [30], [31], [32]. For instance, Zhang et al. indicated that there was no significant difference for this variant in the allele and genotype frequencies between CHD and controls [30]. Besides, no biological and functional analyses have been reported about this variant yet. Thus, the 606G>C variant may not contribute significantly to CHD risk, but the result should be treated with caution because of the low power obtained from our sample size.

To explore the sources of heterogeneity, we further conducted stratified and sensitivity analyses for 63A>G variant. Stratified analyses by the types of CHD and sample size suggested that heterogeneity only presented in the VSD and small-sample-size subgroups, while heterogeneity was effectively removed after deletion of the study by Shi et al. in the sensitivity analysis. Interestingly, the study by Shi et al. was included in the VSD and small-sample-size subgroups, implying that the heterogeneity found in the two subgroups might result from this study. Under review of this report, Shi et al. proposed that 63A>G variant was not significantly associated with ASD, but with VSD. Individuals carrying G allele had a 4.32 - fold increased risk of VSD than those with A allele, which did not conform to other studies. But positive result was still observed after removal of the study conducted by Shi et al., indicating the relatively stability of current meta-analysis. Besides, marginal statistical significance was presented in the sensitivity analysis when several studies were omitted, which was probably caused by limited data and modest effect of this variant.

Some limitations of this meta-analysis should be acknowledged. First, there is evidence that the roles of variants in *NKX2-5* gene may differ across racial backgrounds, while the current meta-analysis was specifically based on the Chinese population, baffling the generalization of our conclusion to other ethnic populations. Second, the sample size of our meta-analysis was relatively small, especially for *NKX2-5* 606G>C. Besides, all included studies were retrospectively designed. Thus, additional large-scale and well designed studies will be required to further confirm our results. Third, we only considered the allelic model in current study because of limited data. Other genetic models should be taken into account to validate our results. Fourth, some heterogeneous natures of studies, including different phenotypes of CHD, female/male ratios and match conditions of control group probably affected our results. However, we were unable to perform further analyses due to lack of detail data.

In summary, our meta-analysis helped for clarifying the discrepancies of genetic studies into associations of *NKX2-5* 63A>G and 606G>C variants with CHD and revealed that the 63A>G, but not the 606G>C was significantly associated with the risk of CHD in the Chinese population. Although the summary risk for developing CHD with the variants of *NKX2-5* gene may be small, CHD occurs in high incidence in China and even a small increase in risk will translate to a large number of potential CHD cases.

## Supporting Information

Figure S1 **The funnel plot for** ***NKX2-5*** **63A>G.**
(TIF)

Figure S2 **The funnel plot for** ***NKX2-5*** **606G>C.**
(TIF)

Checklist S1 **The PRISMA 2009 Checklist.**
(DOC)
