# Supplementary figures and images for: Associations between Two Genetic Variants in NKX2-5 and Risk of Congenital Heart Disease in Chinese Population: A Meta-Analysis
Source: PLoS One. 2013 Aug 2;8(8):e70979. doi: 10.1371/journal.pone.0070979 (PMC3732287; doi:10.1371/journal.pone.0070979)

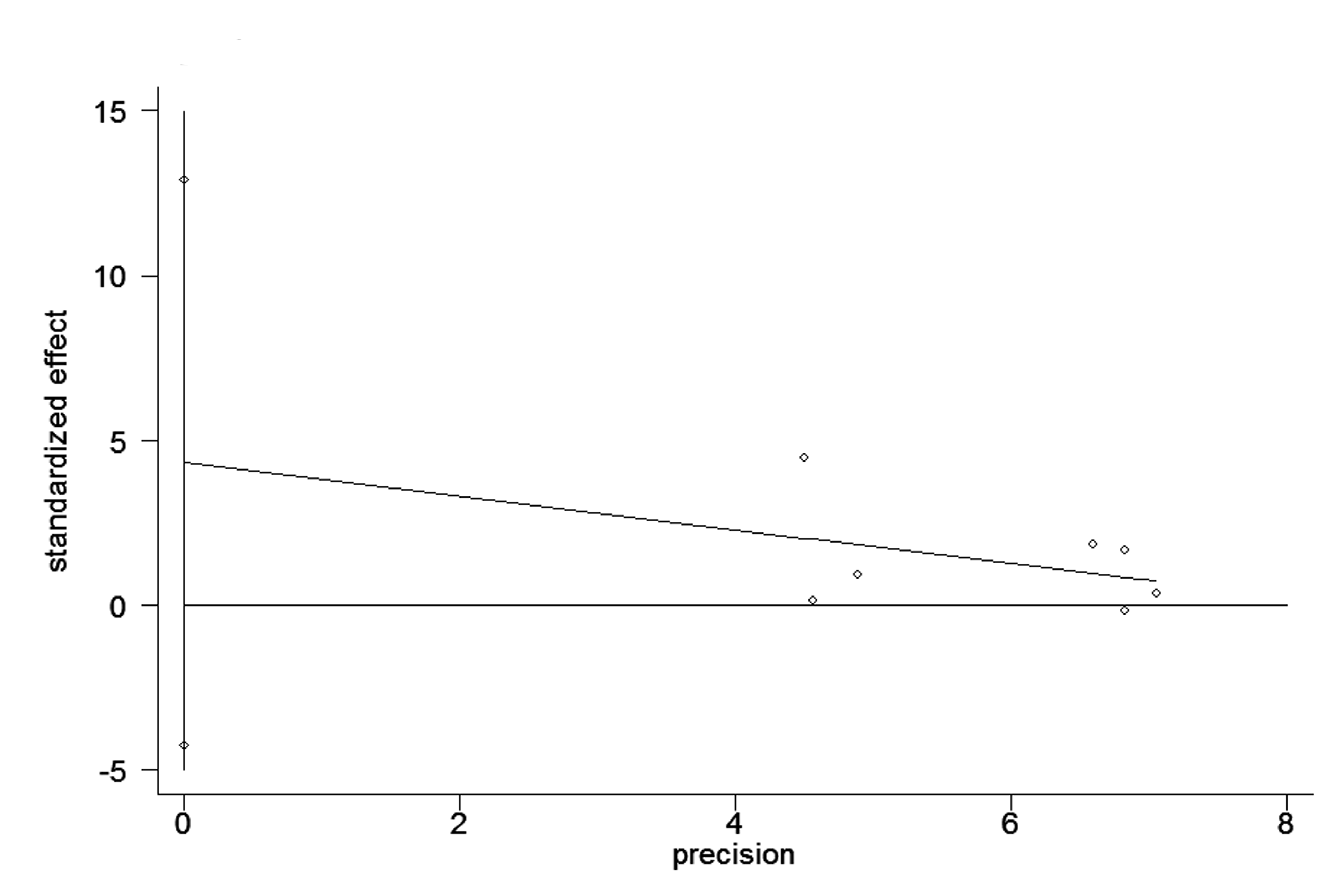

Supplement: Figure S1 — The funnel plot for NKX2-5 63A>G. (TIF) [file pone.0070979.s001.tif]

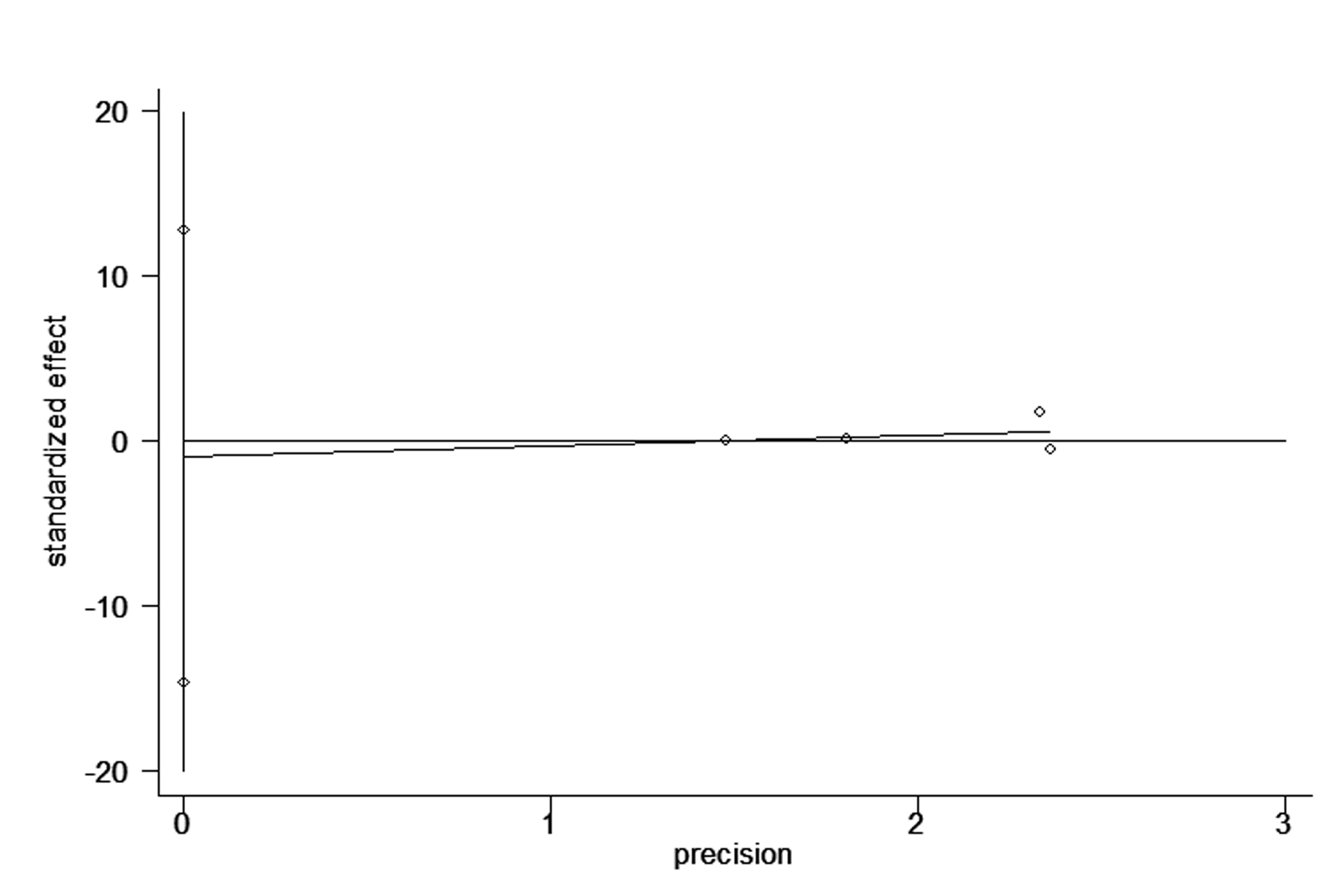

Supplement: Figure S2 — The funnel plot for NKX2-5 606G>C. (TIF) [file pone.0070979.s002.tif]
